# The role of exercise in post-traumatic stress disorder: clinical evidence, sleep, cognition, and psychotherapy augmentation

**DOI:** 10.3389/fpsyt.2026.1889239

**Published:** 2026-08-19

**Authors:** Yuyin Li, Shuyi Li

**Affiliations:** 1 Department of Physical Education, Dankook University, Yongin-si, Republic of Korea; 2 School of Physical Education, Shenyang Sport University, Shenyang, China

**Keywords:** adjunctive therapy, cognition, exercise, post-traumatic stress disorder, psychotherapy augmentation, sleep

## Abstract

Post-traumatic stress disorder (PTSD) constitutes a profoundly incapacitating psychiatric disorder distinguished by the presence of intrusive recollections, heightened arousal, disruptions in sleep patterns, cognitive impairments, and diminished quality of life (QoL). Notwithstanding the advancements achieved in pharmacological treatments and trauma-oriented psychotherapeutic interventions, a considerable number of patients encounter persistent residual symptoms, underscoring the necessity for supplementary therapeutic modalities. This review scrutinizes the nascent role of physical exercise as an adjunctive treatment for PTSD, concentrating on clinical evidence pertinent to the alleviation of symptoms, regulation of sleep, enhancement of cognitive function, and augmentation of the efficacy of trauma-focused psychotherapeutic interventions, particularly exposure-based therapies. Empirical data derived from randomized controlled trials and preliminary studies suggest that modalities such as aerobic exercise, high-intensity interval training, resistance training, and comprehensive exercise regimens may contribute to reductions in the severity of PTSD symptoms, as well as associated anxiety, depression, hyperarousal, and insomnia. Furthermore, exercise interventions have been associated with improvements in cardiorespiratory fitness, mindfulness, interoceptive awareness, and overall psychological QoL across a variety of populations, including veterans, refugees, law enforcement personnel, military service members, and adolescents. Mechanistically, physical exercise appears to augment neuroplasticity through transient elevations in brain-derived neurotrophic factor, modulation of autonomic regulatory functions, and facilitation of fear extinction learning processes. Significantly, aerobic exercise in conjunction with exposure-based psychotherapy has shown promising potential for enhancing long-term clinical outcomes. Collectively, the existing body of evidence suggests that exercise may represent a feasible, safe, and biologically plausible adjunctive intervention for PTSD; however, larger multicenter trials are necessary to establish optimal exercise prescriptions and elucidate the underlying neurobiological mechanisms.

## 1 Introduction

Post-traumatic stress disorder (PTSD) constitutes a severe psychiatric disorder that manifests subsequent to exposure to traumatic occurrences, including military combat, interpersonal violence, natural calamities, accidents, or life-threatening medical conditions. PTSD impacts millions of individuals globally and signifies a significant public health challenge due to its chronic nature, high rates of psychiatric comorbidity, compromised social functioning, and increased healthcare demand. Epidemiological research reveals that the lifetime prevalence of PTSD ranges from approximately 3% to 8% within the general population. However, substantially higher prevalence rates have been documented among high-risk populations, including military veterans (approximately 11%–30%), refugees (up to 40%), and survivors of interpersonal or sexual violence, among whom prevalence estimates often exceed 30% (1–4). In addition to the characteristic symptoms of intrusive thoughts, avoidance strategies, hyperarousal, and adverse changes in cognition and affect, PTSD is significantly correlated with sleep disturbances, cognitive impairment, depression, substance use disorders, cardiovascular ailments, metabolic irregularities, and diminished quality of life (QoL). Recent evidence further suggests that these comorbid conditions substantially contribute to functional disability, increased healthcare utilization, and elevated all-cause morbidity among individuals with PTSD (2, 5–7).

The multifaceted ramifications of this condition underscore that PTSD transcends its classification as merely a psychiatric disorder and is increasingly recognized as a systemic disorder. PTSD is characterized by interconnected alterations across multiple physiological domains, including neurobiological abnormalities (e.g., hippocampal, amygdalar, and prefrontal cortical dysfunction), neuroendocrine dysregulation involving the hypothalamic–pituitary–adrenal axis (HPA), autonomic nervous system imbalance, and chronic low-grade inflammatory activation (8, 9). Notwithstanding the progress made in pharmacological and psychotherapeutic modalities, the management of PTSD continues to present significant challenges. Trauma-focused psychotherapeutic interventions, encompassing cognitive-behavioral therapy (CBT), prolonged exposure therapy, and eye movement desensitization and reprocessing, are recognized as first-line therapeutic options and exhibit efficacy in alleviating symptom intensity (10). Nevertheless, treatment response remains heterogeneous, and a substantial proportion of patients continue to experience residual symptoms or fail to achieve remission. These limitations suggest that the complex and multifactorial pathophysiology of PTSD is not yet fully understood and may not be adequately addressed by existing therapeutic approaches alone. Consequently, there is increasing interest in complementary interventions capable of targeting both the psychological and neurobiological dimensions of PTSD (7, 11–13). Nevertheless, adherence to treatment protocols and rates of therapeutic response frequently remain suboptimal (14, 15). A considerable proportion of patients discontinue psychotherapy prematurely due to multiple factors, including treatment-related emotional distress associated with trauma recollection, symptom severity, comorbid psychiatric conditions, practical barriers to treatment engagement, and individual differences in treatment acceptability and readiness (11, 16, 17). Furthermore, residual symptoms often endure, even following ostensibly successful interventions, particularly in the realms of insomnia, cognitive dysfunction, emotional dysregulation, and social impairment (10, 18–20). Pharmacological strategies for PTSD primarily include selective serotonin reuptake inhibitors, particularly sertraline and paroxetine, which are currently recommended as first-line pharmacotherapies in several clinical guidelines. Other pharmacological approaches, including serotonin-norepinephrine reuptake inhibitors, prazosin for trauma-related nightmares, and various off-label agents, have also been investigated. However, overall treatment effects remain modest, and these interventions are frequently associated with incomplete remission, adverse effects, treatment discontinuation, and inconsistent long-term efficacy (21–23).

Importantly, pharmacotherapy in isolation may not adequately address the broad range of physiological alterations that have been implicated in PTSD, including changes in neuroplasticity, inflammatory signaling, autonomic regulation, and metabolic functioning. Although the precise etiopathogenesis of PTSD remains incompletely understood, growing evidence suggests that these biological processes may contribute to symptom persistence and reduced resilience following trauma exposure (12, 24, 25). These limitations have engendered a growing interest in adjunctive interventions that possess the capacity to complement conventional therapeutic modalities while addressing both psychological and physiological dimensions of PTSD. In the present review, the term “adjunctive therapy” primarily refers to the use of exercise alongside established evidence-based PTSD treatments, including pharmacotherapy and psychotherapy, with the aim of enhancing overall treatment outcomes. However, because a substantial proportion of the available literature has evaluated exercise as a stand-alone intervention, such studies were also included to provide a comprehensive overview of the therapeutic potential of exercise in PTSD. Throughout this review, we explicitly distinguish between exercise delivered as an adjunctive strategy and exercise implemented as an independent intervention. Adjunctive therapies hold particular significance as PTSD is increasingly acknowledged as a disorder characterized by the interplay between brain and body, rather than merely a psychological affliction (12, 13, 26). Chronic alterations in the HPA axis, together with changes in hippocampal and prefrontal cortical functioning, heightened amygdala reactivity, mitochondrial dysfunction, and inflammatory signaling, have been proposed as potential mechanisms underlying PTSD and are thought to contribute to symptom persistence and reduced resilience following trauma exposure (12, 24). Consequently, interventions that concurrently modulate neurobiological and behavioral pathways may provide complementary therapeutic benefits alongside established pharmacological and psychotherapeutic treatments. In this framework, exercise has emerged as a promising non-pharmacological strategy for the management of PTSD.

Physical exercise exerts widespread effects on neural and physiological systems that have been discussed as potentially contributing to the pathophysiology of PTSD. Regular physical activity has been associated with favorable changes in neuroplasticity, hippocampal neurogenesis, monoaminergic neurotransmission, inflammatory signaling, and HPA axis regulation (27, 28). Furthermore, exercise has been associated with increases in brain-derived neurotrophic factor (BDNF), improved cerebral perfusion, and favorable changes in mitochondrial function, all of which may contribute to improvements in cognitive processes and emotional regulation. From a behavioral standpoint, physical activity may mitigate avoidance behaviors, bolster self-efficacy, enhance sleep quality, and promote social interaction, thus simultaneously addressing multiple fundamental characteristics of PTSD (29). Notably, exercise is comparatively accessible, economically viable, and adaptable to various demographic groups, rendering it a compelling adjunctive therapeutic approach in both clinical and community contexts. For the purpose of elucidation, the exercise modalities examined within this review encompass a variety of distinct categories of physical activity. Aerobic exercise denotes continuous rhythmic movements that predominantly enhance cardiorespiratory fitness, exemplified by activities such as ambulation, jogging, cycling, or swimming. Resistance training comprises exercises specifically devised to augment muscular strength and endurance via the application of external resistance, which may include free weights, resistance bands, or weight training apparatus. High-intensity interval training (HIIT) is characterized by the alternation of short, vigorous exercise bouts with intervals of active recuperation or rest. Furthermore, integrative exercise programs may amalgamate aerobic, resistance, mindfulness-based, and flexibility elements to concurrently address multiple physical and psychological domains pertinent to PTSD (27, 30).

Although numerous prior systematic reviews have scrutinized the relationship between physical activity and mental health or investigated exercise interventions within populations afflicted by PTSD, significant knowledge deficiencies persist. Previous reviews have predominantly concentrated either solely on symptom alleviation or on particular cohorts such as military veterans, frequently neglecting the integration of clinical evidence with mechanistic understandings pertaining to sleep, cognitive processes, and the augmentation of psychotherapeutic interventions (30, 31). Moreover, a considerable proportion of existing reviews were executed prior to the recent proliferation of randomized controlled trials that assess aerobic exercise, resistance training, yoga-based movement modalities, and integrative rehabilitation programs for individuals with PTSD. The current body of literature has frequently conceptualized exercise primarily as a lifestyle intervention rather than comprehensively evaluating its potential role as an evidence-informed therapeutic strategy, including its use as an adjunctive approach to enhance psychotherapeutic engagement and address the multifaceted dysfunctions associated with PTSD.

Furthermore, limited attention has been devoted to understanding the interplay between exercise-induced neurobiological changes and the efficacy of trauma-focused psychotherapy, notwithstanding the burgeoning body of evidence indicating that physical activity may enhance emotional processing, extinction learning, and cognitive flexibility, thereby amplifying therapeutic engagement and retention. Consequently, the present review is imperative to furnish a contemporary and integrative synthesis of the extant literature scrutinizing exercise both as an adjunctive and stand-alone intervention for PTSD, with a particular focus on clinical evidence, disturbances in sleep, cognitive impairments, and the enhancement of psychotherapeutic modalities. Instead of merely cataloging exercise-related studies, this review undertakes a critical analysis of the variability within intervention protocols, methodological constraints, mechanistic pathways, and the translational significance of prevailing findings. By integrating neurobiological mechanisms with clinical outcomes, this review seeks to clarify whether exercise should be considered solely as supportive care or may represent an important component of multimodal treatment strategies for PTSD.

Particular emphasis is placed on elucidating deficiencies in the extant evidence concerning exercise intensity, modality, duration, patient adherence, and personalized treatment methodologies, which remain insufficiently explored in the present scholarly discourse. In conclusion, PTSD persists in exerting a substantial psychological, neurological, and societal load notwithstanding the availability of current therapeutic interventions. Although traditional pharmacological and psychotherapeutic approaches are effective for many individuals, a considerable proportion of patients continue to experience residual symptoms or incomplete remission. Furthermore, growing evidence suggests that PTSD involves complex interactions among psychological, neurobiological, and physiological processes, raising the possibility that multimodal interventions targeting both behavioral and biological pathways may provide additional therapeutic benefits.

## 2 Neurobiological basis of exercise in PTSD

### 2.1 PTSD pathophysiology

PTSD is increasingly conceptualized as a multifaceted psychiatric disorder that may involve dysregulation across several neurobiological systems implicated in stress responsiveness, emotional processing, cognitive functioning, and sleep regulation (12, 13). Exposure to traumatic events has been associated with enduring alterations within the HPA axis, autonomic nervous system, immune pathways, and neural circuits involved in fear processing and memory (12, 13, 32). Neuroimaging investigations have consistently reported alterations in the amygdala, hippocampus, and prefrontal cortex, which may contribute to hyperarousal, intrusive memories, emotional dysregulation, and impaired fear extinction (12, 13, 32). Furthermore, PTSD has been associated with increased inflammatory activity, oxidative stress, sleep disturbances, and cognitive impairments that may exacerbate symptom severity and diminish overall QoL (32). Recent findings indicate that physical exercise may concurrently address several of these pathological mechanisms by influencing stress hormone levels, promoting neuroplasticity, modulating inflammatory processes, and enhancing cognitive performance (26). Consequently, comprehending the neurobiological foundations of PTSD is crucial for clarifying how exercise might function as a complementary therapeutic approach.

#### 2.1.1 HPA axis dysregulation

Dysregulation of the HPA axis has been proposed as one of the central neurobiological features of PTSD (33–35). Individuals diagnosed with PTSD often demonstrate modifications in cortisol release patterns, heightened sensitivity to negative feedback mechanisms, and dysregulated activity of glucocorticoid receptors (25, 36). Prolonged exposure to traumatic stressors may alter communication pathways among the hypothalamus, pituitary gland, and adrenal cortex, resulting in maladaptive neuroendocrine responses. Several studies have reported diminished basal cortisol levels, coupled with exaggerated catecholamine release, which may be associated with heightened states of hypervigilance, anxiety, and the persistence of fear memories among individuals with PTSD. Furthermore, sustained dysfunction of the HPA axis has been suggested to negatively influence hippocampal integrity, emotional regulation capacities, and immune system functioning. Exercise has been posited as a modulator of HPA axis activity by facilitating the normalization of cortisol rhythms and diminishing neuroendocrine activation related to stress (37). Engagement in regular physical activity may enhance resilience to stressors and mitigate maladaptive physiological reactions in response to trauma-related cues. Thus, modulation of the HPA axis may represent one of the mechanisms through which physical exercise could contribute to alleviating PTSD symptoms.

#### 2.1.2 Autonomic dysfunction

Dysfunction of the autonomic nervous system is frequently observed in individuals diagnosed with PTSD, manifesting as an augmented sympathetic activation coupled with diminished parasympathetic activity. Individuals suffering from PTSD often present with an increased resting heart rate, diminished heart rate variability, pronounced startle reactions, and elevated catecholamine concentrations (38, 39). Accumulating evidence suggests that these physiological alterations may contribute to persistent hyperarousal, emotional dysregulation, sleep disturbances, and increased cardiovascular risk (40, 41). Prolonged sympathetic overactivation has also been proposed to interfere with emotional processing and reinforce maladaptive fear responses. Similarly, reduced vagal tone and impaired parasympathetic regulation may limit an individual’s ability to recover from stress exposure, thereby perpetuating physiological arousal and anxiety symptoms. Engagement in exercise training has been demonstrated to improve autonomic regulation by attenuating sympathetic activity and enhancing parasympathetic modulation. Aerobic exercise, in particular, has been associated with improvements in HRV and cardiovascular resilience (42). Regular physical activity may therefore contribute to restoring autonomic balance, although the magnitude of these effects appears to vary across studies and exercise modalities (43). Through these mechanisms, consistent participation in physical activity may alleviate symptoms of hyperarousal and promote improved emotional regulation in individuals with PTSD. Consequently, autonomic modulation has been proposed as one of the potential neurobiological targets of exercise-based interventions for PTSD.

#### 2.1.3 Neuroinflammation and oxidative stress

Increasing empirical evidence suggests that neuroinflammation and oxidative stress are integral components in the pathophysiological mechanisms underlying PTSD. The exposure to traumatic stress triggers the activation of immune pathways and facilitates the secretion of pro-inflammatory cytokines (44). Several studies have reported elevated concentrations of inflammatory markers, including interleukin-6, tumor necrosis factor-α, and C-reactive protein, in individuals with PTSD, although findings are not entirely consistent across studies (24, 44). Prolonged inflammatory activity may disrupt neuronal functioning, alter neurotransmitter systems, and contribute to emotional dysregulation and cognitive impairment. Furthermore, PTSD has been associated with increased production of reactive oxygen species and impaired antioxidant defense mechanisms, potentially resulting in oxidative damage to lipids, proteins, and deoxyribonucleic acid (DNA). Oxidative stress has been proposed as one mechanism that may contribute to hippocampal dysfunction and neurodegenerative processes, thereby exacerbating memory deficits and anxiety-related symptoms (45). However, the precise contribution of oxidative stress to PTSD pathophysiology remains an active area of investigation. Physical exercise exhibits anti-inflammatory and antioxidant properties through modulation of cytokine expression and enhancement of endogenous antioxidant defense systems. Regular physical activity has been associated with reductions in systemic inflammatory markers and improvements in mitochondrial function, which may help protect neural tissue from stress-related damage (46). Nevertheless, the extent to which these exercise-induced biological adaptations directly mediate clinical improvements in PTSD has yet to be fully established (47, 48). Consequently, modulation of inflammatory and oxidative pathways may represent one of the potential mechanisms through which exercise could exert therapeutic effects in individuals with PTSD.

#### 2.1.4 Fear circuitry abnormalities

PTSD exhibits a significant correlation with anomalies in neural circuits that govern fear processing, emotional regulation, and the consolidation of memory. Empirical evidence from functional neuroimaging investigations has revealed heightened activity in the amygdala, juxtaposed with diminished activity in the medial prefrontal cortex and the hippocampus (49, 50). However, the extent and nature of these alterations may vary according to trauma type, symptom profile, and methodological differences across studies (32, 51). Hyperactivity of the amygdala has been proposed to contribute to exaggerated fear responses and persistent threat perception, whereas impaired regulatory activity within the prefrontal cortex may hinder the extinction of trauma-related memories. Similarly, hippocampal dysfunction may interfere with contextual memory processing and increase susceptibility to intrusive recollections. Altered functional connectivity among these brain regions has been suggested to contribute to emotional dysregulation, hypervigilance, and maladaptive behavioral responses observed in PTSD (49, 50). Engagement in physical exercise may beneficially influence fear-related neural circuitry by promoting neuroplasticity and enhancing functional connectivity within limbic and cortical networks. Experimental and neuroimaging studies suggest that physical activity may increase hippocampal neurogenesis, improve prefrontal cortical functioning, and attenuate stress-related amygdala reactivity (28, 52). Nevertheless, direct evidence linking exercise-induced neural adaptations to improvements in fear circuitry specifically in PTSD populations remains limited and warrants further investigation. Consequently, modulation of fear-related neural circuits has been proposed as one potential mechanism through which exercise could exert therapeutic effects in PTSD.

#### 2.1.5 Sleep dysregulation

Sleep disturbances represent one of the most common and debilitating manifestations of PTSD. Individuals with PTSD frequently experience insomnia, trauma-related nightmares, fragmented sleep, reduced sleep efficiency, and alterations in rapid eye movement (REM) sleep architecture (18, 53). Recent meta-analytic evidence indicates that both subjective and objective sleep disturbances are highly prevalent among individuals with PTSD and may persist even after successful treatment of core PTSD symptoms (54, 55). Sleep dysregulation has been associated with emotional instability, impaired fear extinction, daytime fatigue, and reduced cognitive performance. Hyperarousal and heightened sympathetic nervous system activity are considered important contributors to sleep disruption in PTSD. Moreover, disturbed sleep may contribute to sustained inflammatory activation and alterations in HPA axis functioning, potentially creating a bidirectional cycle that exacerbates psychiatric symptoms (56). Neuroimaging studies further suggest that sleep abnormalities may interfere with memory consolidation and emotional processing, thereby reinforcing trauma-related memories. Physical exercise has been identified as a promising non-pharmacological strategy for improving sleep quality and regulating circadian rhythms. In both clinical and non-clinical populations, regular physical activity has been associated with improvements in sleep efficiency, sleep duration, and insomnia symptoms (57). Furthermore, exercise may reduce anxiety and physiological arousal, thereby indirectly promoting restorative sleep (57). However, the extent to which exercise-induced improvements in sleep directly mediate reductions in PTSD symptom severity remains insufficiently understood and requires further investigation. Consequently, modulation of sleep pathways has been proposed as one potential mechanism through which exercise may contribute to improvements in PTSD symptomatology.

#### 2.1.6 Cognitive impairments

Cognitive deficits are frequently observed in individuals diagnosed with PTSD and may substantially affect occupational functioning, social relationships, and overall QoL. Common cognitive impairments include deficits in attention, executive functioning, working memory, processing speed, and verbal learning (19, 58). Studies suggest that impairments in verbal memory, attention, and executive functioning are among the most consistently reported cognitive abnormalities in PTSD, although considerable interindividual variability exists (19, 58). Prolonged exposure to stress and trauma-related neurobiological alterations may contribute to structural and functional changes within the hippocampus and prefrontal cortex, brain regions that are essential for cognitive processing. In addition, inflammation, oxidative stress, and sleep disturbances have been proposed as factors that may further exacerbate cognitive dysfunction in individuals with PTSD. Cognitive impairments may also interfere with emotional regulation and reduce responsiveness to psychotherapeutic interventions. Physical exercise has been shown to exert beneficial effects on cognitive performance through multiple mechanisms, including increased cerebral blood flow, enhanced synaptic plasticity, and stimulation of neurotrophic signaling pathways. Aerobic exercise has been associated with increased levels of BDNF and may support hippocampal neurogenesis. However, evidence specifically examining exercise-induced cognitive improvements in PTSD populations remains limited and somewhat heterogeneous (59). Nevertheless, these neurophysiological adaptations may contribute to improvements in memory, attention, and executive functioning among individuals with PTSD (60). Consequently, exercise-induced cognitive enhancement may represent an important mechanism facilitating functional recovery and potentially improving therapeutic outcomes.

### 2.2 Mechanisms through which exercise may improve PTSD

#### 2.2.1 BDNF and neuroplasticity

BDNF constitutes a key mediator of neuroplasticity and plays an important role in neuronal survival, synaptic plasticity, learning, and memory processes. Reduced BDNF expression has been reported in some studies of individuals with PTSD and has been associated with impaired hippocampal function, emotional dysregulation, and cognitive deficits, although findings across studies remain somewhat heterogeneous (61–64). Physical exercise is recognized as one of the most potent non-pharmacological stimuli for increasing BDNF availability (61–63). Both acute and chronic engagement in physical activity have been shown to increase circulating BDNF levels and may promote hippocampal neurogenesis and synaptic remodeling (65). These neuroplastic adaptations have been proposed to contribute to improvements in emotional resilience, memory consolidation, and adaptive stress responses in individuals with PTSD. Moreover, exercise-induced BDNF signaling may facilitate communication between prefrontal and limbic brain regions, thereby potentially supporting emotional regulation and fear extinction processes (64). Although the precise mechanisms remain under investigation, aerobic exercise appears to be particularly effective in stimulating neurotrophic pathways and enhancing cognitive function (66). Consequently, activation of BDNF-mediated neuroplastic mechanisms has been proposed as one of the potential pathways through which physical exercise may alleviate PTSD symptoms and possibly enhance the efficacy of psychotherapeutic interventions.

#### 2.2.2 Fear extinction enhancement

Fear extinction pertains to the attenuation of conditioned fear responses through repeated exposure to trauma-related stimuli in the absence of actual peril. Dysfunctional fear extinction is widely considered to be a core neurobiological feature of PTSD and may contribute to the persistence of avoidance behaviors and intrusive recollections. Physical exercise has been proposed to enhance extinction learning through the modulation of neural circuits involving the hippocampus, amygdala, and prefrontal cortex (67, 68). Experimental and clinical studies suggest that enhanced BDNF signaling and exercise-induced synaptic plasticity may facilitate the consolidation of extinction-related memories (69, 70). Furthermore, engagement in exercise has been associated with reductions in anxiety sensitivity and physiological arousal, which may increase tolerance to trauma-related stimuli during psychotherapy. Emerging empirical evidence indicates that aerobic exercise performed immediately before or after exposure-based interventions may enhance long-term treatment outcomes, although findings remain somewhat inconsistent across studies (71). The activation of catecholaminergic and glutamatergic pathways induced by exercise has also been hypothesized to support learning and memory processes involved in fear extinction. Consequently, physical exercise may represent a promising adjunctive strategy for augmenting trauma-focused psychotherapeutic interventions; however, additional mechanistic and large-scale clinical studies are needed to confirm these effects.

#### 2.2.3 Anti-inflammatory and antioxidant effects

Exercise demonstrates significant anti-inflammatory and antioxidant properties that may mitigate biological alterations associated with PTSD. Consistent engagement in physical activity has been associated with reductions in circulating pro-inflammatory cytokines, while simultaneously promoting the production of anti-inflammatory mediators (72). Moreover, exercise contributes to the enhancement of antioxidant defense mechanisms by increasing the activity of enzymes such as superoxide dismutase, catalase, and glutathione peroxidase (73). These physiological adaptations may contribute to reductions in oxidative damage and improvements in mitochondrial function within neural tissues (74). Accumulating evidence suggests that persistent inflammation and oxidative stress may be involved in hippocampal dysfunction, emotional dysregulation, and cognitive deficits observed in PTSD, although the precise causal relationships remain incompletely understood (24, 44). Therefore, the immunomodulatory effects of exercise have been proposed to confer neuroprotective benefits. Additionally, reductions in inflammatory activity may contribute to improved neurotransmitter homeostasis and decreased stress-related neural toxicity. Moderate-intensity aerobic exercise has been suggested to be particularly effective in modulating inflammatory responses without substantially increasing physiological stress (46, 75). By concurrently influencing immune and oxidative pathways, exercise may contribute to symptom improvement and support long-term neural health in individuals with PTSD; however, additional longitudinal and mechanistic studies are required to clarify these associations.

#### 2.2.4 Sleep modulation

Engagement in physical exercise has the potential to ameliorate symptoms associated with PTSD through the modulation of sleep architecture and the promotion of restorative sleep (76). Empirical evidence indicates that consistent physical activity is associated with reductions in insomnia severity, decreased sleep onset latency, and improvements in sleep efficiency (77, 78). Furthermore, physical exercise has been shown in some studies to promote slow-wave sleep and stabilize circadian rhythms, which are frequently disrupted in individuals with PTSD (57, 79). Improved sleep quality may facilitate emotional regulation, memory consolidation, and recovery following stress exposure. Moreover, the anxiolytic effects of exercise, together with its potential to reduce sympathetic nervous system activation, may contribute to reductions in nighttime hyperarousal and trauma-related nightmares. It has also been proposed that improvements in sleep quality may indirectly contribute to reduced inflammatory activity and more adaptive HPA axis functioning, thereby supporting broader neurobiological recovery processes. However, the causal pathways linking exercise, sleep improvements, and PTSD symptom reduction remain incompletely understood. Both aerobic exercise and mind-body interventions, such as yoga, have been associated with improvements in sleep-related outcomes in trauma-exposed populations (80). Consequently, sleep modulation through exercise has been proposed as one potential mechanism linking physical activity to reductions in PTSD symptom severity and improvements in overall QoL, although further mechanistic research is warranted.

#### 2.2.5 Exercise-induced cognitive enhancement

Exercise-induced cognitive enhancement has the potential to improve functional outcomes in individuals diagnosed with PTSD. Engaging in physical activity has been shown to increase cerebral blood flow, promote angiogenesis, and stimulate the release of neurotrophic factors involved in learning and memory processes (52, 81). Moreover, exercise has been associated with improvements in executive function, attention, processing speed, and working memory, potentially through structural and functional adaptations within the hippocampus and prefrontal cortex (28, 66). These neurocognitive improvements may help individuals with PTSD to regulate emotions more effectively, engage more fully in psychotherapeutic interventions, and reduce trauma-related cognitive distortions. Additionally, physical activity has been associated with reductions in fatigue, depressive symptoms, and stress reactivity, which may indirectly support cognitive functioning. Aerobic exercise, as well as integrated physical–cognitive training programs, has been suggested to be particularly beneficial for enhancing neurocognitive performance (82). However, evidence regarding the magnitude and durability of cognitive benefits in PTSD remains limited and somewhat inconsistent, partly because of heterogeneity in study populations, intervention protocols, and cognitive assessment methods. Enhanced cognitive functioning may contribute to improved treatment adherence and facilitate recovery in social and occupational domains. Consequently, exercise-induced cognitive enhancement has been proposed as one potential therapeutic mechanism that may complement established PTSD interventions and support long-term psychological resilience.

## 3 Exercise interventions and PTSD symptom reduction

**Figure 1:**
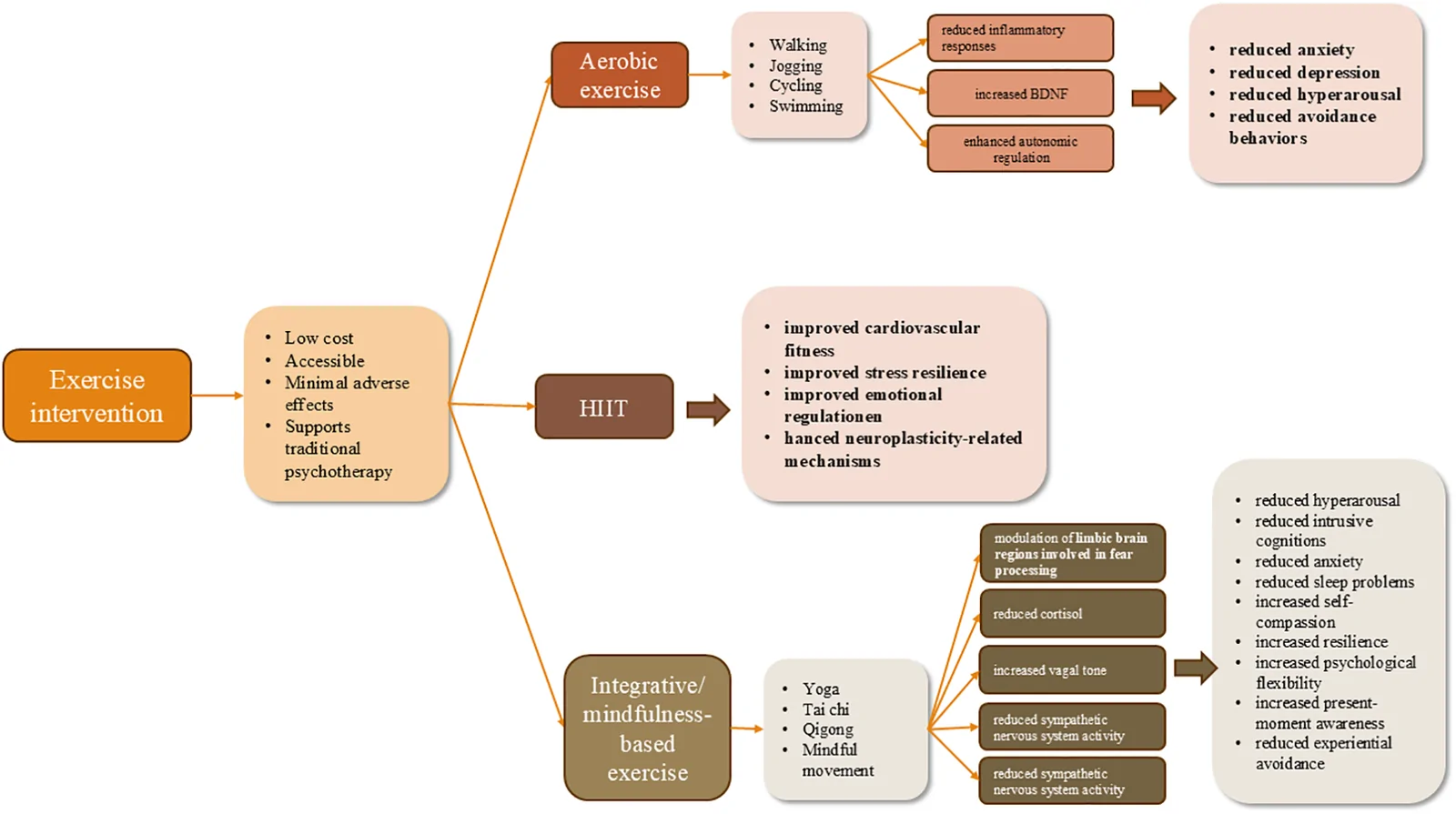
Exercise interventions and PTSD symptom reduction. Exercise is presented as a complementary intervention that may reduce PTSD symptom severity through biological, cognitive, and psychological pathways. Aerobic exercise and HIIT may improve autonomic regulation, neurotrophic signaling, and inflammatory balance, whereas integrative and mindfulness-based exercise may additionally enhance parasympathetic activity, vagal tone, emotional regulation, and psychological flexibility. In special populations such as veterans, group-based integrative exercise combining aerobic, resistance, and mindfulness components has also been evaluated for effects on PTSD symptoms and quality of life.

Exercise interventions have garnered considerable scholarly interest as supplementary methodologies for alleviating symptoms of PTSD and enhancing psychological well-being. Consistent engagement in physical activity has been shown to mitigate hyperarousal, anxiety, depressive manifestations, sleep disturbances, and cognitive impairments frequently encountered in PTSD-affected individuals (29, 70). Furthermore, exercise may improve emotional regulation and resilience through the modulation of neurobiological pathways that encompass neuroplasticity, inflammation, autonomic equilibrium, and regulation of stress hormones. Both aerobic and integrative modalities of exercise have exhibited considerable therapeutic efficacy within populations exposed to trauma. Notably, exercise interventions are typically characterized by low financial cost, accessibility, and a profile of minimal adverse effects when contrasted with pharmacological therapies. Moreover, exercise may enhance adherence to psychotherapeutic interventions by diminishing avoidance behaviors and fostering self-efficacy. While exercise is not recognized as an independent treatment modality for PTSD, an expanding body of evidence advocates for its role as a complementary intervention capable of improving overall therapeutic outcomes and QoL for individuals affected by this condition (29, 83). The neurobiological mechanisms through which exercise may influence PTSD are complex and involve interactions among neuroendocrine, autonomic, inflammatory, and neural systems. Although many of these mechanisms remain under investigation, accumulating evidence suggests that exercise may affect several pathways implicated in PTSD pathophysiology. **Figure 1** summarizes the principal mechanisms that have been proposed to mediate the beneficial effects of exercise in PTSD, including modulation of the HPA axis, autonomic regulation, neuroplasticity, inflammatory signaling, sleep regulation, cognitive function, and fear extinction processes.

### 3.1 Aerobic and HIIT interventions

Aerobic exercise and HIIT represent two of the most thoroughly investigated exercise modalities for the management of PTSD. Engaging in aerobic activities, including but not limited to walking, jogging, cycling, and swimming, has been correlated with significant reductions in symptoms of anxiety, depression, hyperarousal, and avoidance behaviors among individuals exposed to trauma (29). These beneficial outcomes may be attributable to enhanced autonomic regulation, increased production of BDNF, and diminished inflammatory responses. HIIT, defined by brief intervals of high-intensity exercise followed by periods of recovery, has surfaced as a potentially effective intervention due to its efficiency and pronounced physiological benefits. This form of training may contribute to improvements in cardiovascular fitness, resilience to stress, and emotional regulation while concurrently activating neuroplasticity-related mechanisms (84). A subset of studies suggests that engaging in exercise at elevated intensities may elicit more pronounced anxiolytic and antidepressant effects in comparison to moderate-intensity exercise, potentially due to the fact that high-intensity exercise provokes more substantial elevations in BDNF, cardiorespiratory fitness, and neuroendocrine adaptations pertinent to emotional regulation (61, 65). Moreover, the experience of exercise-induced physiological arousal may enhance an individual’s capacity to endure bodily sensations associated with anxiety and hyperarousal, thus facilitating interoceptive processing (70). Nonetheless, the intensity of exercise should be tailored to the individual, as excessive physiological activation may elicit discomfort or exacerbate symptoms in certain individuals diagnosed with PTSD. Furthermore, mind-body interventions that integrate mindfulness elements, such as yoga or comprehensive exercise programs, may serve as appropriate alternatives for individuals who exhibit heightened sensitivity to physiological arousal, given that these methodologies may foster self-regulation, interoceptive awareness, and emotional regulation (85, 86).

### 3.2 Integrative and mindfulness-based exercise

Integrative and mindfulness-based exercise modalities, encompassing yoga, tai chi, qigong, and mindful movement practices, have exhibited advantageous effects in individuals diagnosed with PTSD. These methodologies amalgamate physical exertion with breath control, corporeal awareness, meditative practices, and emotional self-regulation techniques. Mindfulness-based exercise may alleviate hyperarousal, intrusive cognitions, anxiety, and disruptions in sleep patterns by fostering parasympathetic activation and mitigating excessive sympathetic nervous system activity (86). Specifically, yoga interventions have been correlated with enhancements in emotional regulation, interoceptive awareness, and symptomatology associated with trauma. Such benefits may be facilitated through augmented vagal tone, diminished cortisol levels, and the modulation of limbic brain regions implicated in the processing of fear. Additionally, integrative exercise may contribute to the enhancement of self-compassion, resilience to stress, and psychological flexibility, which are pivotal for the recovery from trauma. Furthermore, mindfulness-oriented practices can serve as a complement to psychotherapy by augmenting present-moment awareness and diminishing experiential avoidance (85, 87). Consequently, integrative exercise modalities emerge as promising adjunctive interventions for the management of PTSD and the promotion of holistic recovery.

### 3.3 Exercise in special populations

A randomized controlled trial investigated the impact of a 6-week HIIT regimen on sleep quality among women exhibiting probable PTSD. A cohort of thirty women (PDS-5≥28) demonstrating suboptimal sleep quality were allocated to either the HIIT intervention or a waitlist control condition. Sleep quality, PTSD severity, and anxiety levels were assessed at baseline and during several follow-up evaluations, while high-frequency heart rate variability (HF-HRV) was measured both prior to and following the intervention. The findings revealed a statistically significant interaction between group and time concerning sleep quality (P<0.001), with the HIIT group displaying an improvement from 11.27 ± 2.55 to 5.60 ± 2.03, in contrast to a minimal alteration within the control group (9.47 ± 2.83 to 8.23 ± 2.39), resulting in a substantial effect size (Hedges’ d=-1.57). Mediation analyses suggested that the observed reductions in anxiety and hyperarousal were significant explanatory factors for the enhancements in sleep quality, whereas changes in HF-HRV did not serve as significant mediators (88) (**Table 1**). In addition, another randomized clinical trial aimed to evaluate the efficacy of an Integrative Exercise (IE) program in comparison to a psychoeducation control group among Veterans diagnosed with PTSD. A total of 84 participants who fulfilled the criteria for PTSD as delineated in the Diagnostic and Statistical Manual of Mental Disorders, Fifth Edition (DSM-5) or achieved a score of ≥23 on the Clinician-Administered PTSD Scale for DSM-5 (CAPS-5) were assigned to either intervention group (IE: n=41; REC: n=43) (89). The interventions, spanning six weeks, integrated aerobic, resistance, and stretching exercises alongside mindful breathing (IE) or psychoeducational components (REC). The primary outcomes assessed included PTSD severity as measured by the CAPS-5 and psychological QoL evaluated through the WHOQOL instrument. Both intervention groups exhibited modest yet statistically significant reductions in PTSD symptoms (IE: −8.2 ± 9.9, P<0.001; REC: −7.8 ± 2.0, P<0.001), with no significant differences observed between the groups overall. Additionally, no noteworthy alterations were detected in WHOQOL psychological scores for either group. Nevertheless, within the remote IE subgroup, a higher frequency of session attendance correlated with enhanced PTSD severity (F = 4.62, P = 0.036) and an improved QoL (F = 6.46, P = 0.014) (90).

**Table 1:** Clinical trials investigating stand-alone and usual-care exercise interventions for PTSD symptom severity and psychological outcomes.

Population	Exercise type	Duration	Main outcomes	Findings	Ref
30 women with probable Post-Traumatic Stress Disorder	HIIT cycling exercise	6 weeks	Sleep quality, anxiety, hyperarousal, HF-HRV	↓ Sleep disturbance (d=−1.57); ↓ Anxiety; ↓ Hyperarousal; ↔ HF-HRV	(88)
84 veterans with Post-Traumatic Stress Disorder	Aerobic + resistance + stretching + mindfulness	Not specified	PTSD severity, psychological quality of life	↓ PTSD symptoms in both groups (IE: −8.2; REC: −7.8); ↔ Between-group differences; ↑ Psychological QoL with higher attendance	(90)
60 police officers with PTSD/psychological injury	Supervised mixed exercise sessions	12 weeks	PTSD, depression, anxiety, stress, insomnia	↓ PTSD (d=0.96); ↓ Depression (d=0.71); ↓ Anxiety (d=0.55); ↓ Stress (d=0.69)	(91)
45 male refugees living in a Greek refugee camp	Exercise and sport training program	8 weeks	PTSD, depression, anxiety, QoL, pain, fitness	↓ Anxiety; ↑ Health-related QoL; ↑ Self-perceived fitness; ↑ Handgrip strength; ↑ Cardiovascular fitness; ↓ PTSD symptoms (trend); ↓ Depression (trend)	(92)
47 veterans with Post-Traumatic Stress Disorder	Integrative Exercise (aerobic + resistance + mindfulness)	12 weeks	PTSD severity, psychological and physical QoL	↓ PTSD symptoms (d=−0.90); ↑ Psychological QoL (d=0.53); ↑ Physical QoL (d=0.30)	(93)
81 adults with primary Post-Traumatic Stress Disorder (84% male)	Resistance training + pedometer-based walking + usual care	12 weeks	PTSD, depression, sleep quality, waist circumference, physical activity	↓ PTSD symptoms (−5.4); ↓ Depression; ↓ Waist circumference; ↑ Sleep quality; ↓ Sedentary time	(94)
12 institutionalized female adolescents with Post-Traumatic Stress Disorder symptoms	Moderate-intensity aerobic walking	15 sessions	PTSD, trauma symptoms, anxiety, depression	↓ PTSD symptoms; ↓ Trauma symptoms; ↔ Anxiety; ↔ Depression; Mixed follow-up results	(95)
40 individuals with clinician-diagnosed Post-Traumatic Stress Disorder	High-Intensity Interval Training (HIIT) vs Low-Intensity Training (LIT)	12 days	PTSD symptom severity, depressive symptoms, dissociative symptoms, psychological distress	↓ Clinician-rated PTSD symptoms (d = 0.76); ↓ Self-reported PTSD symptoms; ↓ Depressive symptoms (d = 0.45); ↓ Dissociative symptoms (d = 0.42); ↓ Psychological distress (d = 0.43); ↔ Between-group differences (HIIT vs LIT)	(96)

HIIT, High-Intensity Interval Training; PTSD, Post-Traumatic Stress Disorder; HF-HRV, High Frequency-Heart Rate Variability; QoL, Quality of Life; IE, Integrative Exercise; REC, Recovery Class; CAPS-5, Clinician-Administered PTSD Scale for DSM-5; PCL-C, PTSD Checklist-Civilian Version; WL, Waitlist Control; DSM-IV, Diagnostic and Statistical Manual of Mental Disorders, Fourth Edition. ↑, Increase; ↓, Decrease; ↔, No significant effect / No change.

An open trial conducted an assessment of the preliminary efficacy of the RECONNECT supervised exercise regimen for law enforcement personnel experiencing PTSD or psychological trauma. A total of 60 police officers from New South Wales (35% female; mean age 42.0 ± 8.9 years), the majority of whom had received a clinical diagnosis of PTSD (n=48, 80%), engaged in biweekly exercise sessions over a duration of 3 months. Outcomes, which were evaluated at baseline, 6 weeks, and 12 weeks, encompassed PTSD severity, depression, anxiety, stress, insomnia, and alcohol consumption, and were analyzed utilizing linear mixed models. The findings indicated clinically significant reductions from baseline to week 12 in PTSD symptoms (Cohen’s d=0.96), depression (d=0.71), anxiety (d=0.55), and stress (d=0.69). Nonetheless, the study experienced high rates of attrition and loss to follow-up. Elevated baseline PTSD symptom severity was associated with a greater likelihood of completing post-intervention assessments (91). Another study evaluated the effects of an 8-week exercise and sport training program on mental and physical health outcomes among male refugees residing in a Greek refugee camp who reported clinically relevant PTSD symptoms but had not necessarily received a formal PTSD diagnosis. A total of 45 participants (mean age=25.6 years) successfully completed both baseline and post-intervention evaluations. Outcomes encompassed PTSD symptoms, depression, anxiety, QoL, pain, and fitness-related metrics, which were analyzed utilizing hierarchical regression techniques. Findings indicated that higher participation rates were significantly associated with lower anxiety symptoms, enhancement in health-related QoL, improved self-perceived fitness, and greater physical fitness (as measured by handgrip strength and cardiovascular capacity) at post-intervention. Additionally, trends (although not statistically significant) suggested a decrease in PTSD and depressive symptoms with increased participation levels. Baseline scores were found to be significant predictors for all post-intervention outcomes (92).

The objective of a research was to explore a group-based Integrative Exercise (IE) intervention for veterans afflicted with PTSD, amalgamating aerobic and resistance training with mindfulness methodologies. A cohort of 47 veterans was allocated to either the IE intervention or a waitlist control condition. The duration of the intervention was 12 weeks, comprising three sessions of one hour each per week. Measured outcomes encompassed the severity of PTSD symptoms and QoL, evaluated through standardized assessment tools. In comparison to the control group, the IE cohort exhibited a more pronounced reduction in PTSD symptoms (Cohen’s d=−0.90), a moderate enhancement in psychological QoL (d=0.53), and a lesser improvement in physical QoL (d=0.30). Furthermore, participants indicated a high degree of feasibility and acceptability regarding the program (93). Another trial investigated the impact of incorporating a 12-week exercise regimen into standard care protocols for individuals diagnosed with PTSD. A total of 81 inpatients meeting the DSM-IV-TR criteria for PTSD were allocated randomly to receive either standard care alone (n=42) or standard care supplemented with exercise (n=39). The intervention entailed a regimen comprising three 30-minute resistance training sessions per week, in conjunction with a pedometer-based walking initiative, alongside conventional psychotherapy, pharmacotherapy, and group therapy sessions. The primary endpoint was the severity of PTSD symptoms, as measured by the PTSD Checklist-Civilian version (PCL-C), while secondary endpoints encompassed metrics of depression, sleep quality, levels of physical activity, and various anthropometric assessments. The findings indicated significantly greater reductions in PTSD symptomatology within the exercise cohort when compared to the control group (mean difference=−5.4; 95% CI −10.5 to −0.3; P = 0.04; analyzed n=58). Notable between-group enhancements were also documented in depressive symptomatology, sleep quality, waist circumference, and levels of sedentary behavior (94). A single-case repeated-measures investigation evaluated the impact of a 15-session moderate-intensity aerobic exercise regimen on the manifestations of PTSD, depression, and anxiety symptoms among 12 institutionalized female adolescents. Participants underwent assessments utilizing the Child PTSD Symptom Scale, Trauma Symptom Checklist for Children, Multidimensional Anxiety Scale for Children, and Children’s Depression Inventory across an extended baseline, subsequent to the intervention, and at a one-month follow-up. The data were subjected to analytical scrutiny through ipsative z-score comparisons for individuals exhibiting stable baseline symptoms. The findings revealed significant enhancements in PTSD and trauma-related symptoms following the aerobic walking intervention. Nevertheless, no substantial reductions were detected in anxiety or depressive symptoms, which may be attributed to the low baseline severity observed in a considerable number of participants. Follow-up results were inconclusive, indicating a partial preservation of the observed gains (95). A recent randomized controlled pilot trial by Schoofs et al. (96) evaluated HIIT as a stand-alone intervention in 40 individuals with clinician-diagnosed PTSD. Both HIIT and low-intensity training (LIT) were associated with significant reductions in clinician- and self-rated PTSD symptoms, depressive symptoms, dissociative symptoms, and overall psychological distress; however, no significant between-group differences were observed, suggesting that exercise intensity alone may not determine clinical efficacy in PTSD.

### 3.4 Exercise interventions for PTSD symptoms: evidence and critical appraisal

Across a range of studies, interventions based on exercise (6–12 weeks, extending to a maximum of 3 months) generally yielded improvements in symptoms associated with PTSD, with numerous randomized controlled trials documenting moderate to substantial effects (e.g., d ≈ −0.90 to −1.57; P<0.05–0.001). Nonetheless, the available evidence is constrained by various methodological shortcomings. The sample sizes were predominantly small to moderate (n=12–84 in the majority of randomized controlled trials), which diminishes both statistical power and the generalizability of the findings. A number of studies employed waitlist or inactive control groups, which may increase the potential for expectancy bias. In addition, several studies lacked blinding procedures or reported substantial attrition rates. Furthermore, some studies lacked blinding procedures or reported relatively high attrition rates. The heterogeneity of interventions (including HIIT, resistance training, integrative exercise, and walking) along with variations in intensity and dosage further restricts comparability among studies. Non-randomized and pre–post designs introduce potential selection and maturation biases, thereby undermining causal inferences. Certain outcomes (for instance, HF-HRV mediation and QoL) demonstrated null or inconsistent findings across studies, suggesting that the evidence regarding these outcomes remains inconclusive and that the underlying mechanisms require further investigation. Despite the encouraging reductions in symptoms, the overall certainty of the evidence remains low to moderate because of methodological heterogeneity, relatively short follow-up periods, and the frequent reliance on self-reported outcome measures.

## 4 Exercise, sleep, and cognitive function in PTSD

While section 3 focused primarily on the effects of exercise on overall PTSD symptom severity, the present section specifically examines evidence regarding three clinically important domains frequently impaired in PTSD: sleep, cognitive function, and fear learning and emotional processing. These outcomes are considered separately because disturbances in sleep and cognition substantially contribute to functional impairment and may respond differently to exercise interventions.

### 4.1 Effects of exercise on sleep disturbances in PTSD

Physical exercise has been increasingly investigated as a strategy to improve sleep disturbances in individuals with PTSD, a population in which insomnia, nightmares, and poor sleep quality are highly prevalent. Clinical studies suggest that regular exercise interventions may improve subjective sleep quality, reduce insomnia severity, and enhance overall sleep-related quality of life in individuals with PTSD and trauma-related symptoms (57, 77, 97, 98). Consistent engagement in physical activity has been correlated with a decrease in insomnia intensity, a reduction in sleep onset latency, an improvement in sleep efficiency, and an augmentation of slow-wave sleep (57, 77). Although the underlying mechanisms remain incompletely understood, exercise-induced improvements in sleep may be related to changes in circadian regulation, autonomic balance, and reductions in hyperarousal (see Section 2). A pilot randomized investigation examined the differential effects of HIIT compared to low-to-moderate-intensity exercise on sleep disturbances among individuals diagnosed with PTSD. A cohort of 40 participants was randomly assigned to engage in six exercise sessions distributed over a 12-day period. Sleep-related outcomes were evaluated from baseline measurements to 24 days subsequent to the intervention utilizing the Pittsburgh Sleep Quality Index (PSQI), sleep logs, and actigraphy methodologies. The findings indicated significant enhancements in PSQI scores over time within both exercise modalities (HIIT: −2.28, d=0.56; low-to-moderate: −1.70, d=0.49), with no observable differences between the groups. No notable alterations were detected in sleep logs or actigraphy within the entire sample population. In a subsample exhibiting clinically significant sleep disturbances (n=24), improvements in PSQI scores were more pronounced (HIIT: −2.65, d=0.53; low-to-moderate: −2.89, d=0.88), alongside a modest reduction in wake after sleep onset (99). Exercise has the potential to alleviate anxiety, depressive symptoms, and hyperarousal, all of which are factors that exacerbate sleep disturbances in individuals suffering from PTSD. Aerobic exercise and mind-body interventions, including yoga, have demonstrated positive effects on trauma-related sleep disturbances and nightmares (86). Enhanced sleep quality may subsequently promote emotional processing, fear extinction, and resilience against stressors. However, objective improvements in sleep architecture have not been consistently demonstrated across studies, highlighting the need for further research using polysomnographic and actigraphic assessments.

### 4.2 Effects of exercise on cognitive function in PTSD

Exercise has been shown to confer significant advantages for cognitive functioning in individuals diagnosed with PTSD. Neurobiological changes related to trauma often disrupt attention, executive functioning, working memory, processing speed, and learning capabilities (19). Emerging clinical evidence suggests that exercise interventions may improve several cognitive domains in individuals with PTSD or trauma-related symptoms, particularly executive functioning, attention, and memory performance (62, 66, 100). Although the underlying mechanisms remain under investigation, exercise-related cognitive improvements have been hypothesized to involve neuroplastic adaptations, enhanced cerebral perfusion, and neurotrophic signaling pathways, as discussed in Section 2. A randomized controlled trial scrutinized a ten-week co-developed exercise and sports intervention targeting forcibly displaced populations residing within a refugee camp in Greece, with an emphasis on cognitive function and pain-related outcomes. A total of 142 participants (52.8% female) hailing from regions in Southwest Asia and Sub-Saharan Africa were randomly allocated to either the intervention group or a waitlist control group. The outcome measures encompassed attention-based cognitive tasks (Flanker and Oddball paradigms), pain severity as assessed by a visual analog scale, and cardiorespiratory fitness determined through the Åstrand-Rhyming test, all analyzed via structural equation modeling. The results indicated no significant direct effects of the intervention on cognitive performance or pain (P≥0.332). Nevertheless, the exercise group exhibited considerable enhancements in cardiorespiratory fitness (β=0.17, P = 0.010), which correlated with expedited reaction times in cognitive assessments (β=−0.22, P = 0.004). A trend of marginal significance suggested a decrease in pain correlating with increased adherence to the intervention (β=−0.14, P = 0.065) (101) (**Table 2**). Aerobic exercise, in particular, has been identified as especially efficacious in enhancing executive function and attention in populations exposed to trauma. Additionally, exercise may indirectly bolster cognitive performance by alleviating inflammation, oxidative stress, depressive symptoms, and disturbances in sleep. However, findings across studies remain heterogeneous, and not all investigations have demonstrated significant objective improvements in cognitive performance. Enhanced cognitive performance may lead to increased participation in psychotherapy and assist in social and occupational reintegration. Thus, the cognitive enhancements resulting from exercise represent a significant adjunctive approach for addressing neuropsychological deficits associated with PTSD and fostering long-term functional resilience.

**Table 2:** Effects of stand-alone exercise interventions on sleep, cognition, and neuropsychological outcomes in PTSD.

Population	Sleep/Cognitive outcome	Exercise protocol	Assessment tools	Results	Ref
142 forcibly displaced individuals living in a refugee camp (52.8% women)	Cognitive function, attention, pain severity	10-week co-designed exercise and sport intervention	Flanker task, Oddball paradigm, Visual Analog Scale (VAS), Åstrand-Rhyming VO_2_max test	↔ Direct cognitive effects; ↔ Direct pain effects; ↑ Cardiorespiratory fitness; ↑ Attention/reaction time; ↓ Pain severity trend with higher adherence	(101)
40 individuals with Post-Traumatic Stress Disorder	Sleep quality, wake after sleep onset	6 sessions of HIIT or low-to-moderate intensity training within 12 days	Pittsburgh Sleep Quality Index (PSQI), sleep log, actigraphy	↑ Sleep quality in both groups (HIIT: +2.28 PSQI; LMIT: +1.70 PSQI); ↓ Wake after sleep onset in severe sleep subgroup; ↔ Between-group differences	(99)
21 adults with subsyndromal Post-Traumatic Stress Disorder	Affective state, exercise enjoyment, mood-related cognition	Acute session of Moderate-Intensity Continuous Exercise (MICE) or High-Intensity Functional Exercise (HIFE)	Affective state scales, affective valence measures, exercise enjoyment assessment	↑ Exercise enjoyment (HIFE & MICE); ↑ Energy; ↓ Tension; ↓ Tiredness after MICE; ↔/↓ Calmness after MICE & sedentary condition	(102)
35 women with Post-Traumatic Stress Disorder	Fear extinction learning, threat expectancy, fear recall	Moderate-intensity aerobic exercise vs light-intensity control after extinction training	Threat expectancy ratings, Skin Conductance Response (SCR)	↓ Threat expectancy after reinstatement (P = 0.02); ↔ Spontaneous recovery; ↔ SCR measures	(103)
8 military soldiers/first responders (4 PTSD, 4 controls)	Autonomic nervous system response, stress physiology	13-min vigorous boxing session (70–80% HRmax)	HRV, salivary cortisol, salivary C-reactive protein, depression/anxiety/stress scales	↓ HRV up to 48 h post-exercise in PTSD group; ↔ Cortisol; ↔ Inflammatory markers; ↔ Depression/anxiety/stress	(104)
33 adults with Post-Traumatic Stress Disorder	PTSD symptoms, anxiety sensitivity, hyperarousal	Stationary biking aerobic exercise (6 sessions over 2 weeks) with different attentional focus conditions	PTSD symptom scales, Anxiety Sensitivity (AS) measures	↓ PTSD symptoms; ↓ Anxiety sensitivity; ↓ Hyperarousal; 89% showed clinically significant PTSD improvement; ↓ Benefit with interoceptive focus prompts	(105)

PTSD, Post-Traumatic Stress Disorder; HIIT, High-Intensity Interval Training; LMIT, Low-to-Moderate Intensity Training; PSQI, Pittsburgh Sleep Quality Index; VAS, Visual Analog Scale; VO_2_max, Maximal Oxygen Uptake; MICE, Moderate-Intensity Continuous Exercise; HIFE, High-Intensity Functional Exercise; SCR, Skin Conductance Response; HRV, Heart Rate Variability; HPA, Hypothalamic-Pituitary-Adrenal; HRmax, Maximum Heart Rate; AS, Anxiety Sensitivity; CON, Control Condition; EX, Exercise Condition. ↑, Increase; ↓, Decrease; ↔, No significant effect / No change.

### 4.3 Effects of exercise on fear learning and emotional processing in PTSD

Emerging evidence suggests that physical exercise may influence fear learning, emotional regulation, and affective processing, processes that are highly relevant to the pathophysiology and treatment of PTSD. In particular, exercise performed in temporal proximity to trauma-focused interventions has been hypothesized to facilitate fear extinction learning and improve emotional processing, although the underlying mechanisms remain incompletely understood (see Section 2). A crossover randomized controlled trial investigated the immediate psychological responses to a single session of moderate-intensity continuous aerobic exercise (MICE) and high-intensity functional exercise (HIFE) compared with a sedentary control condition in individuals exhibiting subsyndromal PTSD. A total of 21 participants (15 female; mean age 24.7 ± 9.3 years) completed all experimental conditions within a within-subject design. Affective states, including energy, tiredness, tension, and calmness, were assessed before exercise, immediately afterward, and 20 and 40 min post-exercise. Both exercise modalities were perceived as significantly more enjoyable than the sedentary condition. Energy levels increased immediately following exercise, whereas MICE was associated with greater reductions in fatigue and better preservation of calmness during recovery, suggesting potential benefits for emotional regulation (102). Arandomized study evaluated the efficacy of moderate-intensity aerobic exercise administered during the memory consolidation phase following fear extinction learning in female individuals with PTSD. Thirty-five participants completed a 3-day fear-conditioning paradigm involving fear acquisition, extinction, and recall. Following extinction learning, participants were randomly allocated to either moderate-intensity aerobic exercise or a light-intensity control condition. Although no significant group differences were observed for spontaneous recovery or most physiological outcomes, participants in the exercise group demonstrated a significant reduction in reinstatement-related threat expectancy compared with controls (P = 0.02), suggesting that exercise may facilitate selected aspects of extinction retention and emotional learning (103).

An exploratory study examined acute psychological and physiological responses to high-intensity boxing exercise in individuals with and without PTSD. Eight male participants (PTSD: n = 4; trauma-exposed controls: n = 4) completed a 13-min high-intensity boxing protocol. Outcomes included heart rate variability, salivary cortisol, inflammatory biomarkers, and psychological symptoms. Participants with PTSD exhibited substantial reductions in heart rate variability lasting up to 48 h after exercise, indicating prolonged autonomic activation, whereas no consistent changes were observed in psychological symptoms or biomarkers. These findings highlight that high-intensity exercise may not elicit uniform emotional or physiological responses across individuals with PTSD and underscore the importance of individualized exercise prescription (104). A randomized controlled trial investigated whether attentional focus during aerobic exercise influences symptom reduction in individuals with PTSD. Thirty-three participants completed a 2-week stationary cycling program and were randomized to an interoceptive focus condition, a distraction condition, or an exercise-only condition without attentional instructions. All groups demonstrated significant reductions in PTSD symptoms and anxiety sensitivity. However, participants assigned to the interoceptive focus condition experienced significantly smaller improvements in hyperarousal symptoms and anxiety sensitivity. These findings suggest that the manner in which attention is directed during exercise may influence emotional processing and treatment responses in PTSD (105). Collectively, available evidence indicates that exercise may influence emotional regulation, affective processing, and selected aspects of fear extinction in PTSD. Nevertheless, findings remain preliminary, and larger well-controlled trials are required to determine the optimal exercise modalities, timing, and intensity for enhancing emotional processing and fear learning.

### 4.4 Exercise interventions for PTSD: effects, mechanisms, and methodological limitations

**Figure 2:**
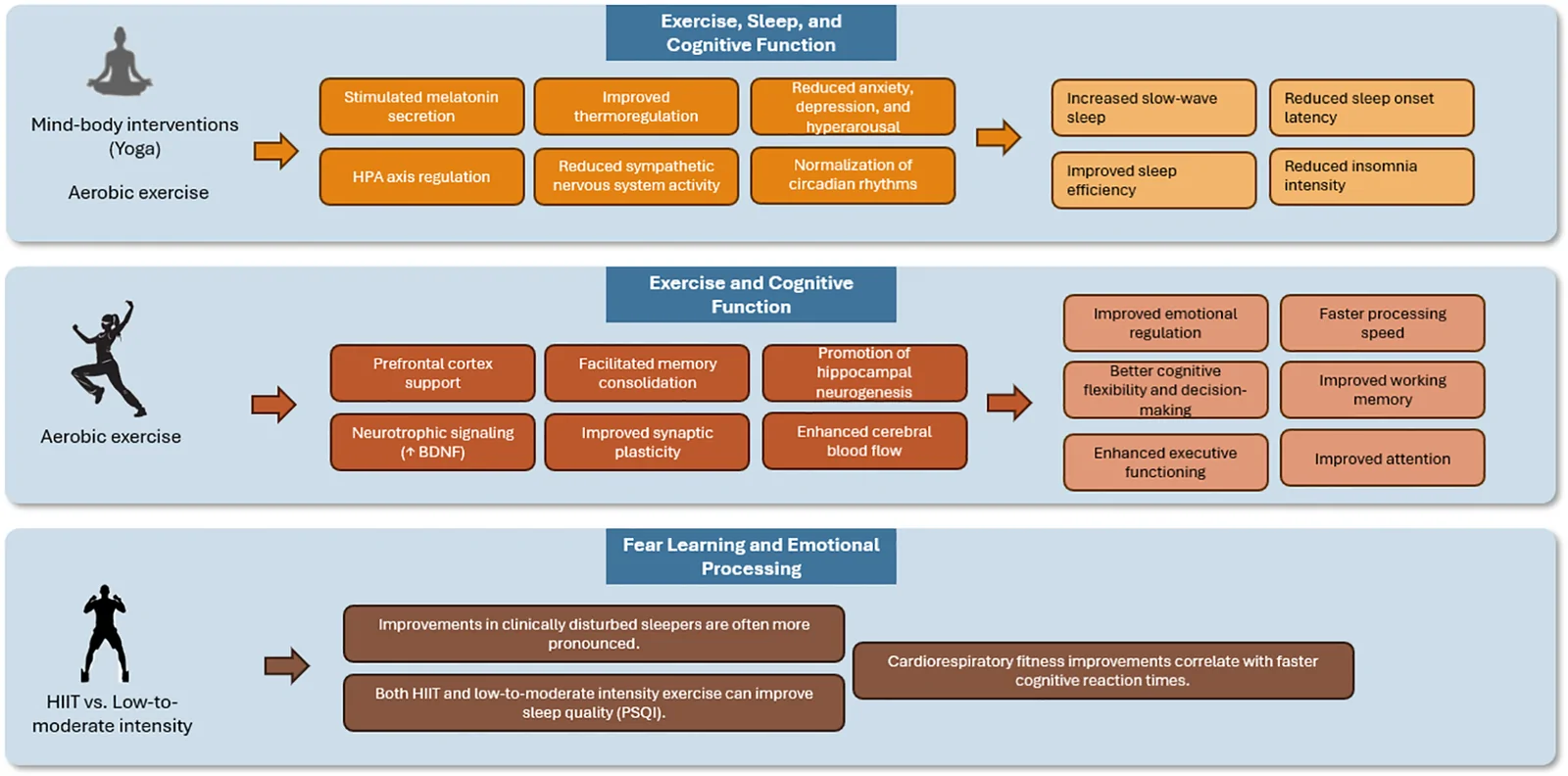
Proposed mechanisms linking exercise and mind–body interventions with sleep, emotional processing, and cognitive outcomes.

In summary, exercise interventions (spanning 6–12 weeks or consisting of acute bouts) have consistently demonstrated reductions in PTSD symptoms, with effect sizes ranging from moderate to substantial (e.g., d ≈ 0.5–1.6) and, in several studies, statistically significant improvements (e.g., *P* < 0.05–0.001). Nevertheless, the overall quality of the evidence remains limited. Sample sizes were frequently modest (n = 8–142, with numerous studies including fewer than 40 participants), although not all studies reported formal *a priori* power calculations for their primary outcomes, making it difficult to determine whether individual trials were adequately powered. A considerable number of trials employed waitlist or inactive control groups, which are appropriate and often necessary for establishing causal relationships; however, such designs may increase susceptibility to expectancy and performance biases when blinding is not feasible. Indeed, the absence of participant and assessor blinding was common across studies. The heterogeneity in intervention duration (ranging from acute exercise bouts to programs lasting up to 12 weeks), together with the limited availability of long-term follow-up assessments, restricts the ability to determine the durability and long-term sustainability of treatment effects. The variability in protocols (including HIIT, resistance training, aerobic cycling, boxing, and integrative exercise), as well as differences in exercise intensity, frequency, and outcome measures, further complicates comparisons across studies. Numerous studies relied predominantly on self-reported measures of PTSD, thereby increasing the risk of measurement bias, and several employed pilot or non-randomized designs, limiting the strength of causal inferences. Overall, although the available findings are encouraging, the current evidence base remains methodologically heterogeneous and provides only low-to-moderate certainty regarding the efficacy of exercise interventions for PTSD (**Figure 2**).

Aerobic exercise and yoga may influence physiological systems through enhanced melatonin secretion, hypothalamic–pituitary–adrenal (HPA) axis regulation, reduced sympathetic nervous system activity, improved thermoregulation, and normalization of circadian rhythms. These adaptations are associated with improved sleep quality, including increased slow-wave sleep, improved sleep efficiency, reduced sleep onset latency, and decreased insomnia severity. Exercise-related neurobiological adaptations—such as increased brain-derived neurotrophic factor (BDNF), enhanced synaptic plasticity, greater cerebral blood flow, and support of hippocampal neurogenesis and prefrontal cortex functioning—may contribute to improved memory consolidation, executive functioning, attention, processing speed, emotional regulation, and cognitive flexibility. Both high-intensity interval training (HIIT) and low-to-moderate intensity exercise have been associated with improved sleep quality and faster cognitive reaction times, with benefits often more pronounced in individuals with clinically disturbed sleep.

## 5 Exercise as an augmentation strategy for psychotherapy

### 5.1 Exercise combined with multimodal PTSD care

An investigation examined the extent to which the BDNF Val66Met polymorphism influences treatment outcomes in individuals diagnosed with PTSD when exposure therapy is administered in conjunction with exercise. A cohort of 85 PTSD sufferers contributed saliva specimens for genotypic analysis and were allocated to a 9-week exposure-based therapeutic regimen augmented with either aerobic physical activity or passive stretching. The participant sample comprised 52 individuals possessing the Val/Val genotype and 33 individuals with the Met genotype. The severity of PTSD symptoms was evaluated both prior to and subsequent to the intervention. Findings revealed that participants with the BDNF Val/Val genotype experienced more pronounced reductions in PTSD symptoms at the conclusion of treatment in contrast to those carrying the Met genotype. Hierarchical regression analyses demonstrated that the most significant alleviations in symptoms were observed in Val/Val individuals who underwent exposure therapy paired with aerobic exercise (106) (**Table 3**).

**Table 3:** Studies investigating exercise as an adjunct to psychotherapy and standard PTSD treatment.

Population	Psychotherapy type	Exercise modality	Biological mechanism	Clinical outcome	Ref
85 PTSD patients (52 Val/Val carriers; 33 Met carriers)	Exposure therapy	Aerobic exercise or passive stretching	BDNF Val allele associated with greater extinction learning and BDNF release	↓ PTSD symptoms greater in Val/Val carriers receiving aerobic exercise + exposure therapy	(106)
130 adults with Post-Traumatic Stress Disorder	Exposure therapy (9 weekly sessions)	Brief aerobic exercise vs passive stretching	Exercise-enhanced extinction learning processes, potentially mediated by BDNF	↓ PTSD severity at 6-month follow-up (mean CAPS-2 difference=12.1; effect size=0.6); ↑ Long-term treatment gains	(107)
72 active-duty military personnel with clinically significant Post-Traumatic Stress Disorder symptoms	Imaginal exposure therapy	Aerobic exercise	Exercise-related modulation of fear/exposure processing	↓ PTSD symptoms over time in all groups; ↔ No superiority of combined exercise + exposure therapy	(108)
103 individuals with chronic whiplash-associated disorder and Post-Traumatic Stress Disorder	Trauma-Focused Cognitive Behavioural Therapy (TF-CBT) vs supportive therapy	Exercise program following psychotherapy	Trauma-processing and rehabilitation mechanisms	↓ PTSD symptoms at 16 weeks with TF-CBT + exercise; ↔ Neck disability differences; ↑ Clinical improvement in both groups	(109)
47 war veterans with Post-Traumatic Stress Disorder symptoms	None (integrative exercise intervention)	Integrative Exercise (aerobic + resistance + yoga + mindfulness-based principles)	↑ Mindfulness (non-reactivity), ↑ interoceptive awareness (body listening), ↑ self-regulation	↑ Mindfulness (d = 0.85); ↑ Interoceptive awareness (d=0.80); ↑ Self-regulation (d=1.05)	(110)
9 adults with Post-Traumatic Stress Disorder	Prolonged Exposure (PE) therapy vs PE + exercise	30-min moderate-intensity treadmill exercise before each PE session	↑ Brain-Derived Neurotrophic Factor (BDNF) and enhanced synaptic plasticity/fear extinction	↓ PTSD symptoms greater in PE+Exercise (d=2.65); ↑ BDNF (d=1.08) compared to PE alone	(70)

PTSD, Post-Traumatic Stress Disorder; IE, Integrative Exercise; PE, Prolonged Exposure therapy; PE+E, Prolonged Exposure plus Exercise; BDNF, Brain-Derived Neurotrophic Factor; HRmax, Maximum Heart Rate; FFMQ, Five-Facet Mindfulness Questionnaire; MAIA, Multidimensional Assessment of Interoceptive Awareness; QoL, Quality of Life. ↑, Increase; ↓, Decrease; ↔, No significant effect / No change.

A single-blind randomized controlled trial investigated the potential of brief aerobic exercise to augment the efficacy of exposure therapy in individuals diagnosed with PTSD. A total of 130 adults, each with a diagnosis of PTSD confirmed by clinical assessment, were randomly assigned to participate in nine weekly sessions of exposure therapy lasting 90 minutes, which included either 10 minutes of aerobic exercise or 10 minutes of passive stretching (n=65 per condition). The severity of PTSD was evaluated utilizing the Clinician-Administered PTSD Scale (CAPS-2) at three distinct time points: baseline, post-treatment, and a subsequent 6-month follow-up. At the 6-month mark, the group engaged in aerobic exercise exhibited significantly greater reductions in PTSD symptoms in comparison to the stretching group (mean difference=12.1; 95% CI 2.4–21.8; P = 0.023), reflecting a moderate effect size (d=0.6). No adverse events were documented throughout the study. Collectively, the findings indicate that brief aerobic exercise may enhance the long-term efficacy of exposure therapy; however, these results are constrained by the specificity of the intervention and the necessity for further extensive replication (107). The aim of a study was to determine whether the incorporation of aerobic exercise augments the efficacy of imaginal exposure therapy for PTSD among active-duty military personnel. A total of 72 participants (predominantly male Army service members aged 22–52 years) exhibiting clinically significant PTSD symptoms (PCL-S≥25) were systematically randomized into one of four experimental conditions: aerobic exercise alone, imaginal exposure therapy alone, a combination of exercise and exposure, or a no-treatment control condition. The severity of PTSD symptoms was longitudinally assessed. The results indicated a statistically significant overall reduction in PTSD symptoms across all experimental groups (P<0.0001); however, no statistically significant differences were observed among the various treatment conditions. The group receiving combined exercise and exposure did not demonstrate superior outcomes compared to either intervention implemented in isolation. Collectively, these findings imply that while there was an observable improvement in symptoms over time, the addition of aerobic exercise to exposure therapy did not yield further advantages within this particular sample. Notably, limitations of the study include a relatively small sample size per group, a pilot study design, and a diminished statistical power to identify between-group differences (108).

A multicenter randomized controlled trial investigated the efficacy of incorporating trauma-focused cognitive behavioral therapy (TF-CBT) alongside exercise in enhancing clinical outcomes for individuals suffering from chronic whiplash-associated disorders (WAD) and posttraumatic stress disorder (PTSD). A cohort of 103 participants (WAD grade II, with a duration exceeding 3 months but less than 5 years) were randomly assigned to receive either TF-CBT in conjunction with exercise (n=53) or supportive therapy (ST) combined with exercise (n=50). Both cohorts underwent a regimen of 10 weeks of psychological intervention succeeded by 6 weeks of physical exercise, with assessments conducted at 16 weeks, 6 months, and 12 months. Application of linear mixed models revealed no statistically significant differences between the groups regarding the primary outcome measure of neck pain-related disability at any assessment interval (e.g., at 16 weeks: mean difference 0.59, 95% CI −4.33 to 5.51). Secondary outcome measures demonstrated a similar pattern of results; however, there were modest enhancements in PTSD symptomatology that favored TF-CBT at the 16-week mark (109).

A trial investigated secondary psychological outcomes associated with a 12-week integrative exercise (IE) program designed for war veterans diagnosed with PTSD. A total of 47 veterans were assigned to either the IE intervention, which incorporated aerobic and resistance training alongside mindfulness-based practices and yoga, or a waitlist control group. Outcomes were evaluated through self-report measures assessing mindfulness, interoceptive awareness, and positive affective states. The results demonstrated substantial intervention effects within the IE group in comparison to the control group, including significant enhancements in mindfulness non-reactivity (d=0.85), interoceptive body listening (d=0.80), and self-regulation (d=1.05). These results imply that integrative exercise may facilitate improvements in cognitive and emotional regulatory mechanisms pertinent to the recovery process from PTSD. Nonetheless, the conclusions drawn are constrained by the limited sample size, dependence on self-reported measures, and the implementation of a waitlist control, which may potentially exaggerate effect size estimates (110). A pilot randomized investigation sought to determine whether acute aerobic exercise could enhance the efficacy of exposure therapy outcomes in individuals diagnosed with PTSD by elevating levels of BDNF. A total of nine participants (eight females; mean age=34 years) were randomly assigned to either prolonged exposure therapy alone (PE) or a combination of exposure therapy and a 30-minute session of moderate-intensity treadmill exercise (PE+E), conducted prior to each therapy session at approximately 70% of the age-predicted maximum heart rate. The primary outcomes assessed included the severity of PTSD symptoms and serum levels of BDNF. The results demonstrated that the PE+E group experienced markedly greater reductions in PTSD symptoms in comparison to the PE group alone (d=2.65) and also displayed an increase in BDNF levels (d=1.08). These findings imply that exercise may enhance exposure-based therapy through neurobiological mechanisms associated with learning and neuroplasticity. Nonetheless, the conclusions drawn from this study are significantly constrained by the exceedingly small sample size, brief duration, and pilot design, necessitating a cautious approach to interpretation and the need for replication in more extensive trials (70).

### 5.2 Exercise as an adjunct in PTSD treatment: evidence and methodological limitations

In totality, exercise-based interventions (ranging from acute bouts to 12-week programs) have consistently demonstrated a reduction in PTSD symptoms and an enhancement of related outcomes, with effect sizes exhibiting a spectrum from small to very large (d≈0.5–2.6; several P<0.05). Nevertheless, the quality of the evidence is significantly hampered by methodological limitations. The sample sizes across studies were predominantly small to moderate (n=8–130), with numerous pilot or subgroup analyses (n<40), thereby constraining statistical power and external validity. The heterogeneity of interventions (including HIIT, aerobic cycling, resistance training, integrative exercise, boxing, and exercise-augmented exposure therapy) diminishes comparability and reproducibility.

A considerable number of studies employed waitlist or passive control groups, which may increase the risk of expectancy and performance biases. Although participant blinding is generally not feasible in exercise intervention studies, several investigations did not report blinding of outcome assessors, thereby increasing the potential for assessment bias. The heterogeneity in intervention durations (ranging from single exercise sessions to programs lasting up to 12 weeks), together with limited follow-up periods, restricts conclusions regarding the durability and long-term sustainability of treatment effects. Several investigations relied on self-reported measures of PTSD or secondary outcomes, thereby increasing the potential for measurement bias. Additionally, exceedingly small genetic or mechanistic subsamples further constrain the ability to make robust inferences.

## 6 Clinical implications and practical considerations

Current empirical findings suggest that physical exercise may serve as a clinically meaningful intervention for PTSD, both as a stand-alone treatment in certain contexts and as an adjunctive strategy alongside evidence-based psychotherapeutic and pharmacological approaches. A range of exercise modalities, encompassing aerobic exercise, resistance training, HIIT, and integrative exercise programs that integrate mindfulness and body awareness, have exhibited positive outcomes on PTSD symptoms, sleep quality, anxiety, depression, and overall psychological well-being. Consequently, exercise prescriptions tailored for individuals with PTSD should be personalized based on the severity of symptoms, physical fitness levels, comorbid conditions, motivational factors, and treatment objectives. Moderate-intensity aerobic exercise emerges as the most consistently well-tolerated and efficacious approach, particularly in alleviating hyperarousal symptoms and enhancing emotional regulation. Nevertheless, HIIT interventions have demonstrated encouraging effects on sleep quality and transient elevations in serum BDNF, indicating potential neuroplastic advantages for select individuals. Despite these encouraging results, adherence continues to pose a significant challenge within populations afflicted by PTSD, due to the potential mitigating effects of avoidance behaviors, diminished motivation, chronic pain, depressive symptoms, and autonomic dysregulation on participation and sustained engagement. Research involving veterans, refugees, and law enforcement personnel indicates that supervised, group-based exercise and integrative rehabilitation programs may enhance adherence by fostering social connectedness, accountability, and enjoyment. It is crucial that exercise interventions be conducted with careful consideration of safety protocols, as intense physical activity may acutely exacerbate autonomic stress responses in certain individuals suffering from PTSD. Consequently, gradual progression, professional oversight, and personalized intensity modifications are imperative. The emergent paradigm of individualized exercise pharmacology may enhance the efficacy of PTSD interventions by integrating biological and psychological factors, which encompass BDNF genetic variations, cardiovascular reactions, sleep irregularities, and initial fitness levels. Furthermore, remote, home-based, and community-centric exercise initiatives present scalable and accessible therapeutic alternatives for populations affected by trauma who face barriers to mental health services. Collectively, these findings substantiate the integration of customized exercise strategies into comprehensive multidisciplinary frameworks for PTSD management.

## 7 Limitations of current evidence

Despite an expanding corpus of empirical evidence endorsing physical exercise as a supplementary intervention for PTSD, several significant limitations undermine the robustness and generalizability of contemporary findings. Numerous investigations are marked by limited sample sizes, especially in the context of pilot studies and proof-of-concept interventions, which constrains statistical power and heightens the susceptibility to both type I and type II errors. Furthermore, the majority of interventions are characterized by their brevity, typically spanning a few sessions to a duration of 6–12 weeks, thereby complicating the assessment of whether the benefits observed are sustainable over extended periods or can facilitate long-term clinical recovery. Another significant constraint is the pronounced heterogeneity of exercise protocols, encompassing variations in intensity (moderate versus HIIT), modality (aerobic, resistance, integrative exercise), frequency, and levels of supervision. This variability complicates the process of making direct comparisons among studies and hinders the formulation of standardized clinical guidelines. Moreover, mechanistic evidence remains limited, with only a fraction of studies assessing biomarkers such as BDNF, cortisol, autonomic function, or inflammatory markers, thereby leaving critical neurobiological pathways inadequately elucidated. Longitudinal follow-up data are also predominantly lacking, which restricts the ability to draw conclusions regarding the sustainability of treatment effects and the prevention of relapse. Lastly, sex-specific differences remain insufficiently examined, notwithstanding evidence indicating disparate physiological and psychological responses to exercise in males and females diagnosed with PTSD. Addressing these limitations is crucial for propelling exercise-based interventions toward evidence-informed clinical practice.

## 8 Future directions

Future investigations concerning the role of exercise as an adjunctive therapeutic intervention for PTSD ought to advance towards more precise and mechanism-oriented methodologies. A significant trajectory in this domain is the establishment of precision exercise medicine, wherein exercise regimens are customized to align with individual clinical profiles, encompassing the severity of symptoms, fitness levels, comorbid conditions, and historical trauma experiences. This tailored strategy has the potential to optimize therapeutic efficacy and enhance long-term compliance. Furthermore, biomarker-guided interventions signify another promising pathway, particularly through the utilization of indicators such as BDNF, cortisol, inflammatory markers, autonomic nervous system activity, and endocannabinoid signaling to inform treatment selection and assess therapeutic responses. The integration of exercise with psychotherapy, notably exposure-based modalities, is also a critical focus, as emergent evidence indicates that exercise may facilitate fear extinction learning and fortify treatment outcomes when implemented as an augmentation strategy. The proliferation of digital and home-based exercise interventions has the potential to significantly enhance both accessibility and scalability, particularly for individuals who encounter obstacles to receiving in-person therapeutic services, including veterans, refugees, and populations affected by trauma in low-resource environments. Concurrently, neuroimaging and molecular research are imperative to more comprehensively illuminate the fundamental mechanisms that underpin exercise-induced enhancements in PTSD, encompassing alterations in hippocampal functionality, neuroplasticity, and the circuitry associated with stress responses. Ultimately, extensive multicenter randomized controlled trials are critical to verify efficacy, establish standardized methodologies, and assess the enduring clinical benefits. Collectively, these prospective avenues of inquiry will facilitate the transition of exercise from a potentially beneficial adjunctive approach into a substantiated element of integrated PTSD treatment. From a translational perspective, future research should also prioritize the development of evidence-based exercise prescription guidelines for individuals with PTSD. Although current evidence does not yet support a standardized exercise prescription for PTSD, existing studies suggest that moderate-intensity aerobic exercise performed 3–5 times per week for approximately 30–60 minutes may provide beneficial effects on PTSD symptoms. Exercise programs should incorporate gradual progression tailored to individual fitness levels and clinical status. In addition, strategies aimed at enhancing adherence—such as supervised sessions, motivational support, and individualized exercise programs—may further optimize treatment outcomes. Nevertheless, additional randomized controlled trials are required to establish precise exercise prescription guidelines regarding frequency, intensity, duration, and progression in PTSD populations.

## 9 Conclusion

The extant body of empirical evidence suggests that physical exercise constitutes a promising adjunctive therapeutic intervention for individuals diagnosed with PTSD. Within a plethora of clinical cohorts, encompassing veterans, refugees, law enforcement officers, military personnel, females with trauma exposure, and adolescents, systematically implemented exercise regimens have been associated with beneficial effects on the severity of PTSD symptoms, quality of sleep, levels of anxiety, depressive symptomatology, and overall psychological well-being. Modalities such as aerobic exercise, HIIT, resistance training, and integrative exercise paradigms that amalgamate mindfulness and bodily awareness appear to confer clinically meaningful benefits, particularly concerning hyperarousal, insomnia, and emotional regulation. A pivotal finding that has arisen from contemporary research is the capacity of exercise to enhance the outcomes of psychotherapeutic interventions, notably those predicated on exposure therapy. Brief bouts of aerobic exercise conducted immediately prior to or subsequent to exposure sessions may facilitate the process of fear extinction learning and may contribute to enduring therapeutic advancements. The observed effects are plausibly mediated through exercise-induced neuroplastic adaptations, which may include transient increases in BDNF, modulation of autonomic nervous system activity, enhancement of cardiovascular regulation, and augmentation of stress resilience. Additionally, physical exercise may facilitate treatment adherence and accessibility by providing a non-stigmatizing, cost-effective complementary strategy. However, the existing literature is characterized by heterogeneity concerning exercise modality, intensity, duration of intervention, and the specific characteristics of patient populations. Numerous studies have been constrained by limited sample sizes, brief follow-up durations, and variability in methodological approaches. Moreover, objective enhancements in sleep quality and cognitive function were not consistently documented, despite improvements in subjective symptomatology. Future large-scale randomized controlled trials are imperative to ascertain optimal exercise prescriptions, identify responders based on biological or psychological profiles, and elucidate the mechanistic pathways that connect exercise to recovery from PTSD. In summary, exercise should not be regarded solely as supportive lifestyle guidance, but rather as a clinically pertinent adjunctive intervention with the potential to enhance both mental and physical health outcomes in individuals suffering from PTSD.
